# High-risk human papillomavirus infection and abnormal cervical cytology among Nepali and Bhutanese refugee women living in eastern Nepal

**DOI:** 10.1186/s12879-017-2186-2

**Published:** 2017-01-14

**Authors:** Madhav P. Bhatta, Derek C. Johnson, Mingma Lama, Shilu Aryal, Pema Lhaki, Sadeep Shrestha

**Affiliations:** 1 Department of Biostatistics, Environmental Health Sciences, and Epidemiology, College of Public Health Kent State University, PO Box 5190, Kent, OH 44242 USA; 2 Department of Public Health & Community Medicine, Tufts University School of Medicine, Boston, MA USA; 3 NFCC, International, Kathmandu, Nepal; 4 Family Health Division, Nepal Ministry of Health, Kathmandu, Nepal; 5 Department of Epidemiology, School of Public Health, University of Alabama at Birmingham, Birmingham, AL USA

**Keywords:** High-risk human papillomavirus, Abnormal cervical cytology, Nepal, Bhutanese refugee, Risk factors, Spousal migration

## Abstract

**Background:**

Cervical cancer is the leading cause of cancer morbidity and mortality among women in Nepal and Bhutan. Data on high-risk human papillomavirus (HR-HPV) infection and cervical abnormalities among Nepali and Bhutanese women are sparse. The objectives of this study were to assess and compare the prevalence of HR-HPV infection and cervical abnormalities among Nepali and Bhutanese women living in Jhapa District in eastern Nepal; and examine the risk factors for HR-HPV infection and cervical abnormalities in those women.

**Methods:**

Study participants were recruited from a women’s health camp organized by NFCC-International, a Nepal-based non-governmental organization, in 2014. Consenting participants were administered a demographic and health questionnaire and cervico-vaginal specimens collected. Both self-collected and clinician-collected cervico-vaginal specimens were tested for HR-HPV infection. Cytologic exam was performed on clinician-collected samples and cervical cytology results were categorized according to the Bethesda classification. A participant was classified as a Bhutanese if they were either born in Bhutan or currently lived in one of the United Nations administered Bhutanese refugee camps in Jhapa; otherwise, the participant was classified as a Nepali.

**Results:**

Of the 647 study participants, 15.9% were Bhutanese women living in refugee camps and the overall age (± standard deviation) was 38.8 ± 8.2 years. The prevalence of HR-HPV infection was 8.9% and abnormal cervical cytology was 7.1% respectively, with no significant difference in HR-HPV positivity (*p* = 0.399) or abnormal cervical cytology (*p* = 0.698) between Nepali and Bhutanese women. Compared to women whose husbands had not migrated for employment, women whose husbands had migrated outside of the district had 3.30 times (95% Confidence Interval [CI]: 1.13–9.64) the odds of being HR-HPV positive and women whose husbands had migrated outside the country had 2.92 times (95% CI: 1.32–6.49) the odds of having abnormal cervical cytology.

**Conclusions:**

HR-HPV positivity and abnormal cervical cytology were similar among Nepali and Bhutanese women. Husbands migrating for employment within or outside the country was a significant risk factor for high-risk HPV infection and cervical cytology, indicating the important role spousal behavior may play in HR-HPV acquisition and cervical abnormalities among these women.

## Background

Cervical cancer is the leading cause of cancer morbidity and mortality among women in Nepal and Bhutan. The International Agency for Research on Cancer estimates of cervical cancer incidence and mortality in 2012 in Nepal were 22.4 and 18.4 per 100,000, and in Bhutan were 17.1 and 11.3 per 100,000 [1]. Cervical cancer is caused by an infection with a “high-risk” human papillomavirus (HR-HPV), which is sexually transmitted and is common among sexually active individuals [2]. Limited data on the prevalence of HR-HPV types in South Asian countries suggest a range of 8–18% in the general female population [3–6]. Specifically, in Nepal, our previous study among women from a rural far-western hilly region found an HR-HPV prevalence of 9.5% [3]. Another study among urban and semi-urban women in the south-central plains region of Nepal reported an HR-HPV prevalence of 8.6% [4]. To the best of our knowledge, there are currently no studies estimating the prevalence of HR-HPV and cervical cancer rates among women in eastern Nepal.

The Bhutanese population consists of three major ethnic groups: the Ngalops, the Lhotshampas, and the Scharchops. The Ngalops are the ethnic majority, and the Scharchops and the Lhotshampas are the two ethnic minority groups. The Lhotshampas are Bhutanese of Nepali origin, whose ancestors migrated to Bhutan, but they share cultural and linguistic similarities with Nepalis. A large proportion of the Lhotshampas in Bhutan fled the country in the early 1990s to escape ethnic and religious persecution and have been living as refugees in eastern Nepal. In the past 8 years, over 100,000 of these Lhotshampas Bhutanese refugees have been resettled in western countries, most notably in the United States [7]. Although over 100,000 have been resettled, about 20,000 still live in refugee camps and the shared socio-cultural similarities between these Bhutanese refugees and the Nepali population in eastern Nepal may indicate a similar risk of HPV and cervical cancer in the two groups. Currently there are no data on HR-HPV prevalence, cervical abnormality, or cervical cancer among Bhutanese refugee women living in Nepal or other countries. Similarly, data on HR-HPV prevalence and cervical abnormality among Bhutanese women in Bhutan are limited as well. A 2014 population-based survey conducted in women from the capital of Bhutan found an HR-HPV prevalence of 18% [6]. However, HPV and cervical cancer data among Bhutanese women in other parts of the country are lacking.

Both Nepali and Bhutanese populations have geographic and ethnic differences that may potentially influence the risk for HPV infections and cervical cancer in these populations. Despite the exodus of a significant proportion of Lhotshampas, or the Bhutanese of Nepali descent from the country, they still make up about a third of Bhutan’s population. Understanding the epidemiology of HPV and cervical cancer abnormalities and the risk factors for cervical cancer in the Nepali and Bhutanese populations is important for prevention of cervical cancer. Thus, the objectives of this study were to assess and compare the prevalence of HR-HPV infection and cervical abnormalities among Nepali and Bhutanese women living in Jhapa District in eastern Nepal; and examine the risk factors for HR-HPV infection and cervical abnormalities among these women. The results of this study have relevance for the understanding of the epidemiology of HPV and cervical cancer among Nepali and Lhotshampa Bhutanese women living in Bhutan, Nepal, and western countries.

## Methods

### Study setting

This study was conducted in Damak Municipality in Jhapa District located in the Terai (lowland) region of eastern Nepal. From the early 1990s Jhapa has been the site of five of the seven United Nations administered refugee camps for Bhutanese refugees in Nepal [8]. The estimated population of the district in 2014 was 855,600. Jhapa is one of the most developed districts in Nepal with high levels of literacy, economic development, transportation infrastructure, and healthcare facilities [9, 10].

### Participant recruitment and consent process

The study participants were recruited from a reproductive health camp organized by NFCC-International, a Nepal-based non-governmental organization, in 2014. Eligibility criteria for participation in the study included: being older than 18 years; able to provide informed consent; not menstruating; having a cervix; currently not being pregnant. Eligible women who were interested in participating in the study were read and explained the informed consent form, and a written informed consent was obtained before enrollment. Women who were not eligible or refused to participate in the study received the same health services as those participating in the study. Institutional review boards from Kent State University, the University of Alabama at Birmingham, and the Nepal Health Research Council approved the study.

### Demographic and health risk questionnaire

The participants in the study were administered a demographic and health risk questionnaire that we developed and used in our previous studies in Nepal. The details of the development and the pilot testing of the questionnaire have been previously described [3, 11, 12]. The survey questionnaire included questions related to socioeconomic indicators, behavioral risk, reproductive health, and migration related factors. The questionnaire was administered by trained, native Nepali speaking research staff.

The demographic characteristics and behavioral risk profile assessed in the study included age, marital status, education, nationality, number of children, previously married or not, age at marriage, husband previously married or not, number of life-time sexual partners, age of sexual debut, current alcohol use, current tobacco smoking, and whether the husband had migrated for work inside Nepal to other districts or outside Nepal. Assessment and classification of nationality was based on answers to two questions: the country of birth or whether they lived in U.N. administered refugee camps in Nepal. A participant was considered a Bhutanese if they either indicated that they were born in Bhutan or currently lived in one of the refugee camps; otherwise, the participant was classified as a Nepali.

### Biological specimens and laboratory analyses

Study protocols for biological sample collection and laboratory analyses have been described in detail previously [3]. During pelvic examinations trained auxiliary nurse midwives collected cervico-vaginal specimens using the APTIMA® Cervical Specimen Collection and Transport (CSCT) kit (Hologic/Gen-Probe, San Diego, CA). Cervico-vaginal samples were transported to the Hologic/Gen-Probe, Inc. laboratory in San Diego for HPV testing. Laboratory testing of HPV was first performed using a generic APTIMA® HR-HPV mRNA (APTIMA® HPV) probe (Hologic/Gen-Probe, San Diego, CA) to detect the presence of E6/E7 mRNA from at least one of 14 different types of HR-HPV (HPV 16, 18, 31, 33, 35, 39, 45, 51, 52, 56, 58, 59, 66, and 68). Samples with a positive HR-HPV test using the generic probe were then were retested with APTIMA® HPV16 18/45 Genotype Assay to detect the presence of HPV16 or HPV18/45 genotypes. Cervico-vaginal samples for cervical cytology were collected in ThinPrep® PreservCyt® medium (Hologic/Gen-Probe, San Diego, CA) and assessed for research purposes with results classified according to the Bethesda criteria. Women with abnormal cervical cytology were referred to local healthcare facilities for further evaluation, testing and clinical follow-up.

### Outcomes and risk factors of interest

HR-HPV infection and abnormal cervical cytology were the two outcomes of interest assessed in this study. HR-HPV infection was defined as testing positive for at least one of the following HPV types: HPV 16, 18, 31, 33, 35, 39, 45, 51, 52, 56, 58, 59, 66, and 68. Cervical cytology was categorized as being either “Normal” or “Abnormal” based on the Bethesda classification system. “Normal” cervical cytology included those samples classified as benign cellular changes, results within normal limits (WNL), atypical squamous cells of undetermined significance (ASCUS), or actinomycosis. “Abnormal” cervical cytology included low-grade squamous intraepithelial lesion (LSIL), high-grade squamous intraepithelial lesion (HSIL), squamous cell carcinoma (SCC), atypical glandular cells of undetermined Significance (AGUS), or atypical squamous cells- cannot exclude high-grade (ASC-H).

Potential risk factors for HR-HPV infection and abnormal cervical cytology assessed in this study included: age (<45 [reproductive age] vs. ≥45 years); formal education (none vs. some); married more than once or ‘previously married’ (yes vs. no); husband married more than once or ‘husband previously married’ (yes vs. no); number of children (≤1, 2, 3 or ≥4); nationality (Nepali vs. Bhutanese); drink alcohol (yes vs. no); smoke any types of tobacco (yes vs. no); husband migrated for work (no, yes- migrated outside the district, or yes-migrated outside the country).

### Statistical analyses

The cervico-vaginal samples were available for HR-HPV testing for 701 women and cervical cytology assessment for 688 women. A total of 679 women completed the survey questionnaires and provided information on nationality. Frequency distribution and proportions were computed for categorical variables and mean (± standard deviation) were computed for continuous variables to describe the demographic and risk behavioral profile for the overall as well as the sample stratified by nationality. Prevalence and 95% confidence interval (95% CI) for HR-HPV infection and cervical cytology were computed for overall and by nationality. The relationship between independent variables described above and the two main outcome variables (HR-HPV infection and abnormal cervical cytology) was assessed using univariable and multivariable logistic regression models. Crude and adjusted Odds Ratio (OR) and corresponding 95% CI and *p*-value are reported. The multivariable logistic regression models included the following independent variables: nationality; age; formal education; and husband migrated for work. SAS version 9.3 (SAS Institute, Cary, NC) was used to perform all statistical analyses.

## Results

### Demographic characteristics and risk behavioral profile

Of the 679 women who completed the survey questionnaires, 574 (84.1%) were Nepali and 105 (15.9%) were Bhutanese. Table 1 provides the demographic and risk factor profile of the participants in the study. The mean age of the sample was 38.8 (±8.2) years, with Nepali women being significantly older than Bhutanese women [39.5 (±8.1) vs. 35.0 (±7.8) years; *p* <0.001]. All the women, except one, were currently married, widowed or divorced, with a mean age at marriage of 19.2 (±4.0) years. Nepali women were significantly more likely to report being currently married compared to Bhutanese women (98.3 vs. 92.8%; *p* = 0.001). They also reported an older age at marriage than Bhutanese women [19.2 (±4.0) vs. 18.0 (±3.9) years; *p* = 0.003]. Bhutanese women were significantly more likely to report being married more than once (or previously married) (8.1 vs. 1.1%; *p* < 0.001). About 11% of the women reported that their husband had been previously married. The mean number of children reported was 2.4 (±1.3). About 29% of the women reported no formal education. There was no significant difference between Nepali and Bhutanese women in regards to husband being previously married, or the level of formal education.

**Table 1:** Demographic Characteristics and Risk Behavioral Profile of Samples of Nepali and Bhutanese Women Living in Eastern Nepal

Characteristics	*n* (%)			
	Overall*	Nepali	Bhutanese	
	679 (100.0)	574 (84.1)	105 (15.9)	*p*- value
Age, years				<0.0001
19–30	24 (3.5)	6 (1.1)	18 (17.4)	
30–34	226 (33.3)	187 (32.6)	39 (37.1)	
35–39	122 (18.0)	104 (18.1)	18 (17.1)	
40–44	115 (16.9)	98 (17.1)	17 (16.2)	
45–69	192 (28.3)	179 (31.2)	13 (12.4)	
Formal education				
None	219 (32.3)	179 (32.2)	40 (38.1)	0.1640
Some	460 (67.5)	395 (68.8)	65 (61.9)	
Marital Status				
Currently married	630 (97.5)	540 (98.4)	90 (92.8)	0.0011
Other^Ɨ^	16 (2.5)	9 (1.6)	7 (7.2)	
Previously married				<0.0001
Yes	14 (2.2)	6 (1.1)	8 (8.1)	
No	634 (97.8)	543 (98.9)	91 (91.9)	
Husband previously married				0.8481
Yes	69 (10.6)	59 (10.7)	10 (10.1)	
No	579 (89.4)	490 (89.3)	89 (89.9)	
Number of children				<0.0001
≤ 1	138 (21.0)	98 (17.6)	40 (40.4)	
2	259 (39.4)	240 (43.0)	19 (19.2)	
3	160 (24.4)	140 (25.1)	20 (20.2)	
≥ 4	100 (15.2)	80 (14.4)	20 (20.2)	
Drink alcohol				0.0010
Yes	55 (8.7)	38 (7.1)	17 (17.4)	
No	576 (91.3)	495 (92.9)	81 (82.7)	
Smoke				0.0326
Yes	60 (9.4)	45 (8.3)	15 (15.2)	
No	579 (90.6)	495 (91.7)	84 (84.8)	
Husband migrated for work				0.0432
No	398 (74.5)	328 (72.6)	70 (85.3)	
Outside the district	24 (4.5)	21 (4.6)	3 (3.7)	
Outside the country	112 (21.0)	103 (22.8)	9 (11.0)	

*Not all values may add to 679 due to missing data; Number of missing values for: Age = 0 (0%); Formal education = 0 (0%); Marital status = 33 (4.9%); Previously married = 31 (4.6%); Husband previously married = 31 (4.6%); Number of children = 22 (3.2%); Drink alcohol = 48 (7.1%); Smoke = 40 (5.9%); and Husband migrated for work = 145 (21.5%)

^Ɨ^Includes single (*n* = 1), divorced/separated (*n* = 6), or widowed (*n* = 9) women

The mean number of life-time sexual partner reported was 1.0, with no significant difference between two groups. The mean age at first sexual debut was 19.3 (±4.1) years with Bhutanese women [18.2 (±4.1) years] reporting a younger age than Nepali women [19.6 (±4.0) years]; a difference that coincided with the difference in the mean age of marriage. About 9% of the women reported drinking alcohol and currently smoking. Bhutanese women were significantly more likely to report drinking alcohol (17.4 vs. 7.1%; *p* = 0.001) and current smoking (15.1 vs. 8.0%; *p* = 0.033) than Nepali women. Over a quarter of the women reported that their husbands had migrated for work, with 21% reporting migration outside the country. Nepali women were almost two-times more likely to reported spousal migration for employment than Bhutanese women (27.4 vs. 14.7%; *p* = 0.04).

### High-risk HPV infection and cervical cytology

In the 713 cervico-vaginal samples available for HR-HPV testing, the prevalence of HR-HPV positivity was 8.4% (95% CI: 6.4–10.5%). When including only the participants who answered the nationality questions (*n* = 641), the prevalence of HR-positivity was 8.9% (95% CI: 6.7–11.1%) with no significance difference in HR-HPV prevalence between Nepali and Bhutanese women (*p* = 0.399). Among HR-HPV positive women, 41.7% were positive for HPV 16, 18 or 45 with no significant difference between the two groups (*p* = 0.850). Among HR-HPV positive women, abnormal cervical cytology was 33.3% compared to 5.0% among HR-HPV negative women (*p* < 0.001).

The prevalence of abnormal cervical cytology in the 640 satisfactory samples (out of 688 total samples) was 7.3% (95% CI: 5.3–9.4%). When including only women with the information on nationality (*n* = 576), the prevalence of abnormal cervical cytology was 7.1% with no significance difference between Nepali and Bhutanese women (*p* = 0.698). There was also no significant difference in cervical cytology results among women with and without the nationality information (results not shown). HR-HPV positivity was strongly associated with abnormal cervical cytology [OR = 9.6; 95% CI: 4.9–14.9]. Table 2 provides details on HR-HPV positivity and cervical cytology data among Nepali and Bhutanese women.

**Table 2:** High-risk Human Papillomavirus (HPV) Infection and Abnormal Cervical Cytology in Samples of Nepali and Bhutanese Women Living in Eastern Nepal

	*n* (%)			
	Overall	Nepali	Bhutanese	*p*- value
High-risk HPV infection (*n* = 641)				0.3993
No	584 (91.1)	496 (91.5)	88 (88.9)	
Yes	57 (8.9)	46 (8.5)	11 (11.1)	
Cervical cytology^Ɨ^ (*n* = 576)				0.6982
Normal	535 (92.9)	458 (92.7)	77 (93.9)	
Abnormal	41 (7.1)	36 (7.3)	5 (6.1)	
Cytology results in detail				0.5387
Normal				
WNL	473 (82.1)	406 (82.4)	66 (80.5)	
ASCUS	62 (10.8)	51 (10.3)	11 (13.4)	
Abnormal				
ASC-H	7 (1.2)	5 (1.0)	2 (2.4)	
AGUS	10 (1.7)	10 (2.0)	0 (0.0)	
LSIL	20 (3.5)	18 (3.6)	2 (2.4)	
HSIL	4 (0.7)	3 (0.6)	1 (1.2)	

^Ɨ^Cervical cytology classification: “Normal” includes: benign cellular changes, *WNL* results Within Normal Limits, *ASCUS* Atypical Squamous Cells of Undetermined Significance, or actinomycosis; “Abnormal” includes: *ASC-H* Atypical Squamous Cells- cannot exclude High-grade, *LSIL* Low-grade Squamous Intraepithelial Lesion, *HSIL* High-grade Squamous Intraepithelial Lesion, *AGUS* Atypical Glandular Cells of Undetermined Significance; and Squamous Cell Carcinoma (none in this sample)

Table 3 presents results of the univariable analyses of the risk factors for HR-HPV positivity and abnormal cervical cytology. In the multivariable analysis, women whose husbands had migrated outside of the districts were 3.30 times (95% CI: 1.13–9.64) as likely to be HR-HPV positive compared to women whose husbands had not migrated for employment (Table 4). Similarly, in the multivariable analysis, women who husbands had migrated outside the country for work had 2.92 times the odds of having abnormal cervical cytology (95% CI: 1.32–6.49) compared to women whose husband had not (Table 4).

**Table 3:** Univariable Analyses of Risk Factors for High-risk Human Papillomavirus (HPV) Infection and Abnormal Cervical Cytology in a Sample of Nepali and Bhutanese Women Living in Eastern Nepal

Risk Factor	High-risk HPV					Cervical Cytology^Ɨ^				
	*n* (%)					*n* (%)				
	Total	Positive	Negative	OR (95% CI)^§^	*p*- value	Total	Abnormal	Normal	OR (95% Cl)^§^	*p*- value
Nationality										
Nepali	542 (84.6)	46 (8.5)	496 (91.5)	1.00		494 (14.2)	36 (7.3)	458 (92.7)	1.00	
Bhutanese	99 (15.4)	11 (11.1)	88 (88.9)	1.35 (0.67–2.70)	0.4000	82 (85.8)	5 (6.1)	77 (93.9)	0.83 (0.31–2.17)	0.6984
Total	641 (100.0)	57 (8.9)	584 (91.1)			576 (100.0)	41 (7.1)	535 (92.9)		
Age, years										
45–69	175 (27.4)	9 (5.1)	166 (94.9)	1.00		151 (26.3)	10 (6.6)	141 (93.4)	1.00	
19–44	464 (72.6)	48 (10.3)	416 (89.7)	2.13 (1.02–4.44)	0.0438	423 (73.7)	31 (7.3)	392 (92.7)	1.12 (0.53–2.33)	0.7725
Total	639 (100.0)	57 (8.9)	582 (91.1)			574 (100.0)	41 (7.1)	533 (92.9)		
Formal education										
None	185 (28.9)	15 (8.1)	170 (91.9)	1.00		162 (28.1)	9 (5.6)	153 (94.4)	1.00	
Some	456 (71.1)	42 (9.2)	414 (90.8)	1.15 (0.62–2.13)	0.6570	414 (71.9)	32 (7.7)	382 (92.3)	1.42 (0.66–3.05)	0.3642
Total	641 (100.0)	57 (8.9)	584 (91.1)			576 (100.0)	41 (7.1)	535 (92.9)		
Current marital Status										
Other*	15 (2.5)	0 (0.0)	15 (100.0)			13 (0.0)	0 (0.0)	13 (100.0)		
Married	595 (97.5)	54 (9.1)	541 (88.7)	**		535 (100.0)	41 (7.7)	494 (92.3)	**	
Total	610 (100.0)	54 (9.1)	556 (90.9)			548 (100.0)	41 (7.5)	507 (92.5)		
Previously married										
No	603 (97.7)	54 (9.0)	549 (91.0)			540 (97.6)	39 (7.2)	501 (92.8)		
Yes	14 (2.3)	1 (7.1)	13 (92.9)	***		13 (2.4)	1 (7.7)	12 (92.3)	***	
Total	617 (100.0)	55 (8.9)	562 (91.1)			553 (100.0)	40 (7.2)	513 (92.8)		
Husband previously married										
No	550 (89.1)	49 (8.9)	501 (91.1)	1.00		490 (88.9)	36 (7.3)	454 (92.7)	1.00	
Yes	67 (10.9)	6 (9.0)	61 (91.0)	1.01 (0.41–2.45)	0.9900	61 (11.1)	4 (6.6)	57 (93.4)	1.13 (0.39–3.29)	0.8228
Total	617 (100.0)	55 (8.9)	562 (91.1)			551 (100.0)	40 (7.3)	511 (92.7)		
Number of children										
≤ 1	130 (20.9)	13 (10.0)	117 (90.0)	1.00		119 (21.2)	14 (11.8)	105 (88.2)	1.00	-
2	242 (38.8)	22 (9.1)	220 (90.9)	0.90 (0.44–1.85)	0.7746	219 (39.1)	13 (5.9)	206 (94.1)	0.47 (0.22–1.04)	0.0637
3	154 (24.7)	11 (7.1)	143 (92.9)	0.69 (0.30–1.60)	0.3907	137 (24.5)	8 (5.8)	129 (94.2)	0.47 (0.19–1.15)	0.0977
≥ 4	97 (15.6)	9 (9.3)	88 (90.7)	0.92 (0.38–2.25)	0.8557	85 (15.2)	6 (7.1)	79 (92.9)	0.57 (0.21–1.55)	0.2700
Total	623 (100.0)	55 (8.8)	568 (91.2)			560 (100.0)	41 (7.3)	519 (92.7)		
Drink alcohol										
No	573 (91.4)	49 (8.6)	524 (91.4)	1.00	-	515 (91.5)	34 (6.6)	481 (93.4)	1.00	
Yes	54 (8.6)	6 (11.1)	48 (88.9	1.34 (0.55–3.28)	0.5258	48 (8.5)	6 (12.5)	42 (87.5)	2.02 (0.80–5.09)	0.1353
Total	627 (100.0)	55 (8.8)	572 (91.2)			563 (100.0)	40 (7.6)	523 (92.4)		
Currently smoke										
No	550 (90.8)	50 (9.1)	500 (90.7)			517 (90.8)	38 (7.4)	479 (92.7)		
Yes	56 (9.2)	2 (3.6)	54 (96.4)	***		47 (9.2)	3 (6.4)	44 (93.6)	***	
Total	606 (100.0)	52 (8.6)	554 (91.4)			564 (100.0)	41 (7.3)	523 (92.7)		
Husband migrated for work										
No	393 (74.6)	30 (7.6)	363 (92.4)	1.00		354 (74.2)	17 (4.8)	337 (95.2)	1.00	
Outside the district	23 (4.4)	5 (21.7)	18 (78.3)	3.36 (1.17–9.69)	0.0248	19 (4.0)	1 (5.3)	18 (87.5)	***	
Outside the country	111 (21.0)	10 (9.0)	101 (91.0)	1.20 (0.57–2.53)	0.6363	104 (21.8)	13 (12.5)	91 (94.7)	2.83 (1.33–6.05)	0.0073
Total	527 (100.0)	45 (8.5)	582 (91.5)			477 (100.0)	31 (6.5)	446 (93.5)		

^Ɨ^Cervical cytology classification: “Normal” includes: benign cellular changes, *WNL* results Within Normal Limits, *ASCUS* Atypical Squamous Cells of Undetermined Significance, or actinomycosis; “Abnormal” includes: *ASC-H* Atypical Squamous Cells- cannot exclude High-grade, *LSIL* Low-grade Squamous Intraepithelial Lesion, *HSIL* High-grade Squamous Intraepithelial Lesion, *AGUS* Atypical Glandular Cells of Undetermined Significance; and Squamous Cell Carcinoma (none in this sample)

^§^OR (95% CI) = Odds Ratio (95% Confidence Interval)

*Other included single, divorced/separated, and widowed women

**OR undefined

***Too few data to estimate an OR

**Table 4:** Multivariable Analyses of Risk Factors for High-risk Human Papillomavirus (HPV) Infection and Abnormal Cervical Cytology in a Sample of Nepali and Bhutanese Women Living in Eastern Nepal

Risk Factor	High-risk HPV (*n* = 527)		Abnormal Cervical Cytology^Ɨ^ (*n* = 476)	
	aOR (95% CI)^§^	*p*- value	aOR (95% CI)^§^	*p*- value
Nationality				
Nepali	1.00	-	1.00	-
Bhutanese	1.31 (0.59–2.92)	0.5046	1.05 (0.34–3.19)	0.9368
Age, years				
45–69	1.00	-	1.00	-
19–44	2.06 (0.84–5.04)	0.1141	0.92 (0.35–2.44)	0.8665
Formal education				
None	1.00	-	1.00	-
Some	0.72 (0.35–1.49)	0.3780	0.93 (0.37–2.30)	0.8662
Husband migrated for work				
No	1.00	-	1.00	-
Outside the district	3.30 (1.13–9.64)	0.0294	1.13 (0.14–9.00)	0.9112
Outside the country	1.14 (0.53–2.48)	0.7346	2.92 (1.32–6.49)	0.0084

^Ɨ^Cervical cytology classification: “Normal” includes: benign cellular changes, *WNL* results Within Normal Limits, *ASCUS* Atypical Squamous Cells of Undetermined Significance, or actinomycosis; “Abnormal” includes: *ASC-H* Atypical Squamous Cells- cannot exclude High-grade, *LSIL* Low-grade Squamous Intraepithelial Lesion, *HSIL* High-grade Squamous Intraepithelial Lesion, *AGUS* Atypical Glandular Cells of Undetermined Significance; and Squamous Cell Carcinoma (none in this sample)

^§^aOR (95% CI) = adjusted odds ratio (95% Confidence Interval); multivariable models included all the variables listed for each outcome

## Discussion

This study, conducted among a community-based sample of Nepali and Bhutanese women living in the eastern Terai region of Nepal, found an HR-HPV prevalence of 8.9%. The observed prevalence of HR-HPV in this study is similar to our previous observation of a 9.6% HR-HPV prevalence in a sample of women from the far-western hilly region of Nepal [3]. Similar prevalence (9.9%) of HR-HPV has been observed in the neighboring Indian state of Uttar Pradesh [13]. The previously mentioned 2014 study from Bhutan reported an overall HPV prevalence of 26%, and HR-HPV prevalence of 18%, in a sample of general population women from the capital city of Thimpu [6]. Any HPV positivity prevalence among the Lhotsampas (Bhutanese of Nepali descent) was reported at 19.8%. While the study did not report HR-HPV separately for the Lhotsampa women, assuming the ratio of HR-HPV positivity to any HPV positivity in this group is similar to the general population in Bhutan, the expected prevalence of HR-HPV among the Lhotsampa women is likely to be around 14%. Among Bhutanese women in our sample, the prevalence of HR-HPV was 11%. However, the small sample size of Bhutanese women in our study and the fact that most of them were originally from the rural south of Bhutan make the comparison of HR-HPV prevalence between our sample of Lhotsampa Bhutanese women with those from the capital of Bhutan problematic. In any case, our study adds to the growing body of literature on the population level data on the HR-HPV distribution among Nepali and Bhutanese women in particular, and South-Asian women in general, which is critical for estimating the cervical cancer risk and targeted intervention efforts locally, and globally among South Asian diaspora.

In addition to the HPV vaccine, primary prevention efforts for HPV infection and cervical cancer focus on the reduction of sexual exposure to HPV through behavioral interventions for the woman and her sexual partner. In our study, we found that almost all the women had one life-time sexual partner—the husband—and the age of sexual debut coincided with the age of marriage. These results on the number of life-time sexual partners, and the age of sexual debut and marriage for Nepali women are consistent with other population-based data [14]. So, for most Nepali women, the husband’s sexual behavior is a key determinant of her risk of HPV acquisition and, thus, the cervical cancer. This observation is supported by our findings that the employment-related migration of the husband within and outside Nepal is associated with an increased risk of HR-HPV infection and cervical abnormalities. We found that women who reported that their husband had migrated outside the district of their residence for employment purposes were almost five times as likely to have an HR-HPV infection compared to women whose husband had no history of employment-related migration. Similarly, women who reported their husband had migrated outside the country for employment were almost three times as likely to have abnormal cervical cytology compared to women with a husband without a history of employment-related migration. These observation have tremendous implications for the primary prevention measures for HPV infection and cervical cancer in Nepal.

Previous studies have shown that men who are engaged in professions that require travel or have migrated for employment have been found to be at a higher-risk of STIs including migrant workers from Nepal to India and other countries [15–17]. Men, when away from their wives or partners for an extended period of time, are likely to engage in high-risk sexual behaviors, including multiple sexual partners and sex without condoms, with other high-risk sexual partners including commercial sex workers. This puts a married woman in Nepal, who may only have one life-time sexual partner, at a heightened risk for HPV and cervical cancer. With over 2 million Nepalis (7% of the total population), primarily men [18], engaged in migrant work within and outside of the country annually, a significant proportion of the women in Nepal are at an increased risk of STI infections, including HPV infection, and cervical cancer. Thus, the behavioral interventions for reducing HPV infection and cervical cancer in Nepal should target husbands and partners as they are likely the primary source of infection for the majority of the women.

We observed that over 7% of the women in the study had abnormal cervical cytology. These women were referred to the regional hospital for further testing, diagnosis, and follow-up treatment and care. Early detection using the cytology smears or non-cytology based screening methods, diagnosis, and treatment, when necessary, of these cytologic abnormalities has been the cornerstone of the cervical cancer prevention over the last several decades globally. The visual inspection with acetic acid (VIA) method is the official protocol of the government for cervical screening in Nepal. However, only a very small proportion of Nepali women get this cervical cancer screening annually. A recent nation-wide survey of women reported that only 13% of women were aware of a cervical cancer screening test and only 5% had ever undergone a cervical cancer screening test [19]. Lack of screening, diagnosis, and treatment for women with abnormal cervical cytology continues to pose a significant barrier for cervical cancer prevention among Nepali women. Along with primary prevention through HPV vaccination and decreasing sexual exposure to HPV infection, the provision of screening with follow-up diagnosis and treatment is vital, if Nepal is to reduce cervical cancer morbidity and mortality.

To the best of our knowledge, this is the first study that has assessed the prevalence of HR-HPV infection and cervical abnormalities among Bhutanese women living in Nepal or western countries. There are about 20,000 Bhutanese refugees still living in Nepal and about 100,000 have been resettled in western countries, most notably in the United States [6]. The findings of the study have implications in cervical cancer prevention among Bhutanese women in Bhutan, Nepal, and western countries. A small study (*n* = 42) conducted among resettled Bhutanese women in the United States found that only 22.2% report ever hearing of a Pap test and 13.9% report ever having one [20]. Moreover, this study also reported that only one in three women perceived themselves to be susceptible to cervical cancer. The results of the current study show that Bhutanese women are at risk of HR-HPV infection and cervical cancer. This highlight the need for increasing awareness in this population regarding cervical cancer as a potential health risk, its cause and the available primary and secondary prevention modalities as first steps towards cervical cancer prevention efforts.

The study results should be interpreted in the context of the strengths and limitations of the study. The sample for the study was recruited from a health camp as a convenience sample. Thus, the Nepali women in the study may not be a representative sample of all the Nepali women living in Jhapa district. Similarly, the Bhutanese women in the study may not a representative sample of the Bhutanese refugee women in the camps. Moreover, the majority of Bhutanese refugees in Nepal have been resettled in western countries, thus, those still remaining in Nepal and participating in the study may suffer from selection bias. However, as the first study among this group to assess HR-HPV infection and cervical abnormalities, the results provide important preliminary information on the cervical cancer risk in this population. As a cross-sectional study, we measured the prevalence of HR-HPV infection, not the life-time exposure or persistence of HPV, which are better predictors of cervical precancerous lesions. However, HPV prevalence data is still a useful proxy for assessing cervical cancer risk, when longitudinal data are not available. Also, our study used an mRNA based test to detect the presence of HR-HPV, a test that detects the expression of genes related to E6/E7. Most population-based studies of HPV prevalence have relied on DNA-based techniques to detect the presence of the HR-HPV virus genome. Thus, the prevalence observed in our study may not be directly comparable to studies using DNA-based techniques.

## Conclusions

The prevalence of HR-HPV and abnormal cervical cytology among Nepali and Bhutanese women in eastern Nepal were similar to the prevalence observed in our previous study conducted in far-western Nepal [3]. While the sexual risk behaviors reported by the women indicated them as being a low-risk for HPV infection, husband’s migration outside the district or the country was a significant risk factor for high-risk HPV infection and cervical cytology. Employment-related migration is a surrogate for high-risk sexual behavior among men. These findings highlight the important role husband’s sexual behavior plays in HPV infection and cervical cancer risk among Nepali and Nepali women. Since the HPV vaccine is not widely available and used in Nepal yet, and millions of women are too old to be vaccinated, an important prevention effort for cervical cancer needs to focus on reducing high-risk sexual behaviors among men. The low level of knowledge regarding cervical cancer cause and prevention methods observed among Nepali and Bhutanese-Nepali women [12, 20] highlight the need for health education to increase the knowledge and awareness of the risk factors for HPV infection and cervical cancer risk.

## Abbreviations

HPV: Human papillomavirus
HR: High-risk
UN: United Nations
US: United States
